# Spontaneous right whole-lung torsion secondary to bronchial carcinoma: a case report

**DOI:** 10.1186/s13019-016-0506-z

**Published:** 2016-07-14

**Authors:** Konstantina Chrysou, Konstantinos Gioutsos, Alexandra Filips, Roger Schmid, Ralph A. Schmid, Gregor J. Kocher

**Affiliations:** Division of General Thoracic Surgery, University Hospital of Bern, Bern, Switzerland; Department of General Surgery, Buergerspital Solothurn, Solothurn, Switzerland

**Keywords:** Spontaneous lung torsion, Whole lung torsion, Pulmonary torsion, Bronchial carcinoma, Lobectomy, Case report

## Abstract

**Background:**

Spontaneous whole lung torsion is an absolut rarity and most cases occur after previous surgery.

**Case presentation:**

We present the case of a spontaneous whole-lung torsion in a 82-year old man. The patient was referred to our thoracic surgery department from the emergency department of a referring hospital with rapidly progressive dyspnea. CT-scan revealed a 180° degree counterclockwise torsion of the entire right lung with complete atelectasis and congestion of the upper lobe as well as pleural effusion. Thoracoscopy confirmed lung torsion and revealed hemorrhagic infarction of the upper lobe. Subsequently thoracotomy and upper lobectomy were performed. Most likely the lung torsion occurred due to a combination of pleural effusion and venous congestion with complete atelectasis of the upper lobe as a result of adenocarcinoma of the upper lobe.

**Conclusions:**

To our knowledge this is the first reported case of a patient presenting with lung torsion as the first symptom of lung cancer. When lung torsion is suspected rapid diagnosis is crucial in order to prevent hemorrhagic lung infarction.

## Background

Pulmonary torsion is a rare event and occurs when a lobe or the entire lung rotates around the bronchovascular pedicle. Most cases occur secondary to thoracic surgical procedures and affect the middle lobe after upper lobe resection. Spontaneous torsions are very rare and may occur due to pneumothorax, pleural effusion, lobar atelectasis or well lobation [1]. Spontaneous torsion of the entire lung is an extreme rarity with only four documented cases in the English literature since 1987 [2–5]. To our knowledge this is the first case of spontaneous lung torsion in combination with pleural effusion and upper lobe atelectasis caused by a primary bronchial carcinoma.

## Case presentation

**Fig. 1:**
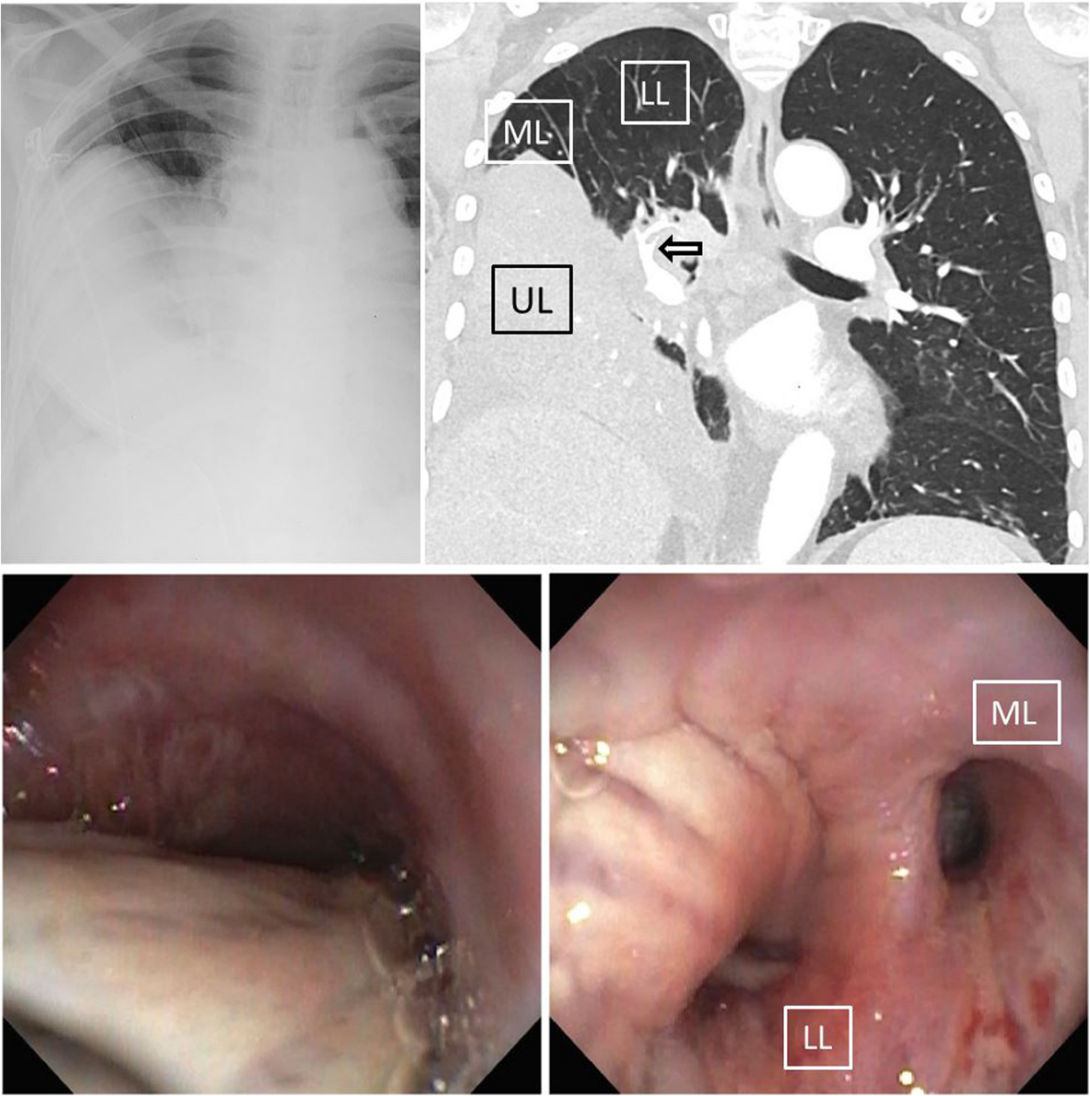
Upper images: Chest X-ray showing right sided lung atelectasis and pleural effusion. On the right: Chest-CT showing downward rotation of the congested and completely atelectatic upper lobe (UL) to the base of the chest cavity with concomitant upward shift of the middle (ML) and lower lobe (LL). Also note the upward torsion of the pulmonary artery to the right lower lobe resulting in a typical ‘whirl sign’ (*arrow*). Lower images: Brochoscopy showing compression/torsion of the right mainstem bronchus (*left image*). Whereas the upper lobe bronchus was completely obstructed, the middle (ML) and lower lobe bronchus (LL) showed rightward torsion resulting in partial obstruction of the corresponding lobes (*right image*)

**Fig. 2:**
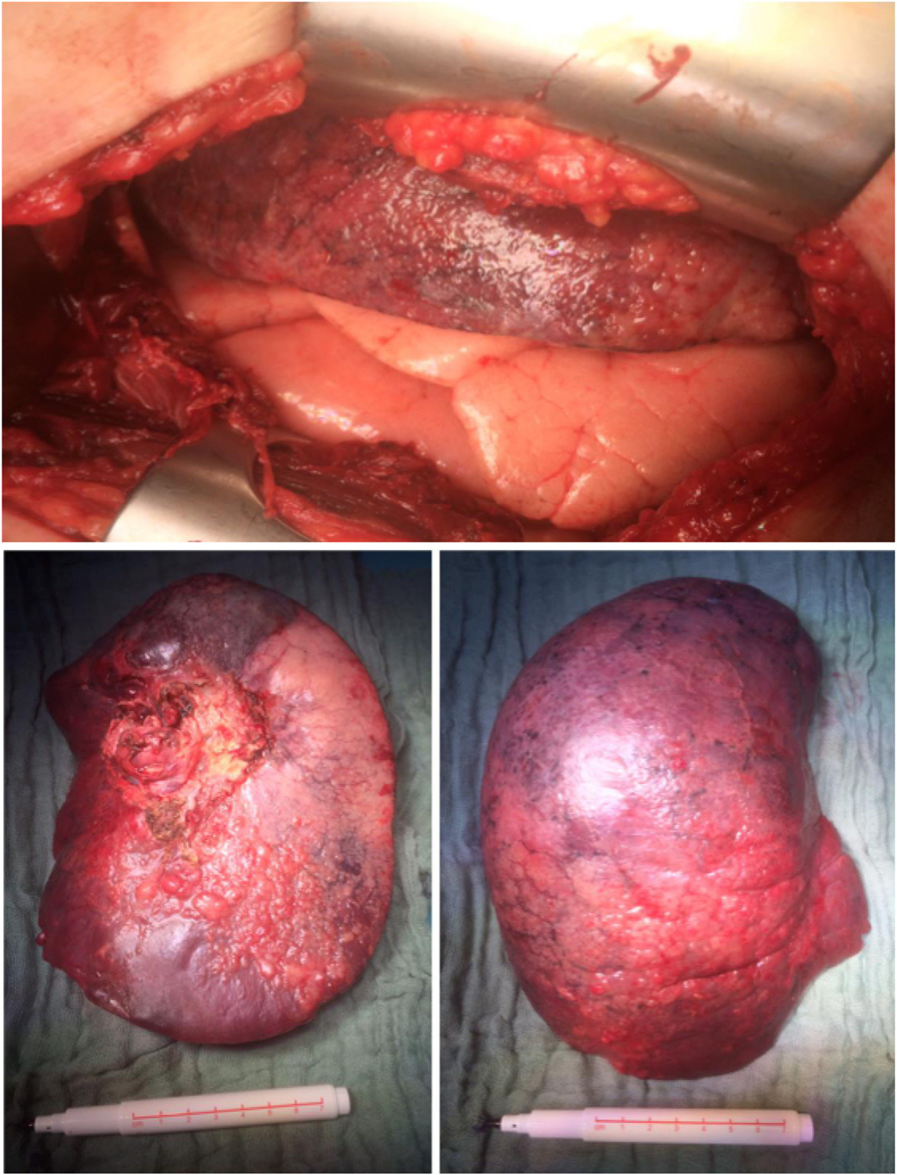
After derotation the upper lobe shows no signs of recovery with complete hemorrhagic infarction compared to the vital middle and lower lobe (*upper image*). Resected congested upper lobe specimen (*below*)

A 82- year old male patient, with the only known comorbidities being hypercholesterolemia and sigmadiverticulosis, presented to a nearby hospital with productive cough and progressive dyspnea in the last 14 days. Chest x-ray showed a right lung opacification with pleural effusion. Subsequent chest CT scan revealed a 180° degree counterclockwise torsion of the whole right lung with partial atelectasis of the lower lobe and middle lobe as well as venous congestion and complete atelectasis of the upper lobe (Fig. 1). The patient was then transferred to our thoracic surgical unit for further treatment. Upon arrival in our department a chest tube was placed on the right side and 1 l of pleural fluid was evacuated. Furthermore bronchoscopy was performed, showing torsion of the right mainstem bronchus with complete obstruction of the upper lobe. The intermediate bronchus showed even more profound rightward torsion with partial obstruction of the middle and lower lobe orifice (Fig. 1). After drainage 12 h later a follow-up CT scan was performed, which showed complete evacuation of the pleural effusion with better ventilation of the middle and lower lobe but persistent lung torsion with suspected pulmonary venous occlusion and venous congestion of the upper lobe. A tumor was not suspected, but could not be excluded due to massive congestion of the whole upper lobe. Therefore the patient was taken to the operating room for diagnostic thoracoscopy. As suspected, the entire right lung was torqued in a 180-degree counterclockwise direction. Since thoracoscopic detorsion could not be achieved and the whole upper lobe showed hemorrhagic infarction, we converted to thoracotomy and performed anatomic resection of the right upper lobe (Fig. 2). The upper lobe vein was centrally compressed by a tumor resulting in venous congestion and finally hemorrhagic infarction of the entire upper lobe. This finding was also confirmed by the pathologist who diagnosed a central bronchial adenocarcinoma in the upper lobe with compression and invasion of the upper lobe vein and additional hemangiosis carcinomatosa. The patient had an uneventful post-operative course and was discharged home in good condition on postoperative day 6. Because of concomitant malignant pleural effusion an adjuvant chemotherapy was recommended.

Unfortunately the patient died 2 months later due to rapid tumor progression.

## Discussion

Spontaneous torsion of an entire lung is an extremely rare condition with this case report being only the 5th reported case since 1987. Furthermore the reported case is the first occurring in a patient with bronchial carcinoma. In the presented case the 180° counterclockwise lung torsion was most likely due to occlusion of the upper lobe vein, resulting in hemorrhagic infarction of the affected lobe. In combination with a large malignant effusion, gravity might have caused the infarcted upper lobe to rotate downwards, whereas the still ventilated middle and lower lobe “floated” upwards on the pleural effusion.

Early recognition and prompt intervention are essential in preventing hemorrhagic infarction or gangrene of the lung, which may finally result in severe sepsis and multiorgan failure. Radiologic diagnosis of lung torsion can be tricky due to the rarity of this condition, especially in patients who have not undergone prior surgery.

## Conclusions

Timely exploratory thoracoscopy and/or thoracotomy are mandatory for rapid recognition and treatment when torsion is radiologically suspected [1]. Whether de-torsion is sufficient or not, which means that resection has to be performed, depends on intraoperative findings
